# Sexual Consent Across Diverse Behaviors and Contexts: Gender Differences and Nonconsensual Sexual Experiences

**DOI:** 10.1177/08862605211044101

**Published:** 2021-10-08

**Authors:** Malachi Willis, Rebecca Smith

**Affiliations:** 1 University of Glasgow, UK; 2 University of Greenwich, London, UK

**Keywords:** sexual consent, sexual diversity, gender, nonconsensual, sexual assault

## Abstract

Sexual consent refers to people’s internal willingness to engage in sexual activity with another person—as well as their external communication of that willingness. Internal and external sexual consent can vary by type of sexual behavior; however, previous research on sexual consent has primarily only assessed “typical” sexual behaviors such as genital touching, oral sex, and vaginal–penile sex without providing further context or acknowledging people’s sexual diversity. Therefore, we provided an initial account of people’s sexual consent—and lack thereof—for a broader array of sexual behaviors and contexts in which they occur. Using an online cross-sectional survey of participants in the United Kingdom and the United States (*N* = 658, 50.5% women), we examined event-level internal and external sexual consent for 20 sexual behaviors or contexts. Women reported significantly lower levels of sexual consent feelings than men for 12 of the 20 sexual behaviors and lower levels of active consent communication for 7 of them. Almost a third of participants (31.0%) had experienced at least one of the listed sexual behaviors against their will. Of those, participants on average reported nonconsensual experiences with 3.1 of the 20 types of sexual behavior listed, ranging from 1 to 11. More women reported at least one nonconsensual experience with one of the sexual behaviors assessed compared with men (47.9% versus 22.3%, respectively). We discussed several behavior-specific findings regarding sexual consent and the lack thereof. We also made recommendations for initiatives aimed at promoting healthy sexual consent practices: embrace sexual diversity, emphasize sexual agency, and encourage active consent communication.

## Introduction

Sexual consent is complex and contextual. Evidencing this, research suggests that sexual consent varies by type of behavior; however, previous studies on sexual consent have primarily investigated only a small selection of behaviors (e.g., genital touching, oral sex, vaginal sex; Jozkowski & Peterson, 2013; Willis, Hunt, et al., 2019). Limiting research to this set of “typical” sexual behaviors without providing further context does not reflect people’s sexual diversity. For example, Herbenick et al.’s (2017) study found that sexual behaviors commonly involve various body parts (e.g., anal stimulation or penetration), combinations of people (e.g., two people of the same gender, group sex), substance use (e.g., alcohol, other drugs), enhancers (e.g., sex toys, role playing), or technology (e.g., sexting, phone/video sex). These diverse and potentially stigmatized variations of sexual behavior are rarely, if ever, assessed in studies on sexual consent. By emphasizing a select few sexual behaviors, research and education initiatives may neglect the importance of promoting the need for consent across all types of sexual activity—not only those most likely to be defined as “quintessential” sex (e.g., vaginal–penile sex; Opperman et al., 2014). Further highlighting the value of evaluating people’s sexual consent to a broader range of sexual experiences, recent evidence demonstrated that contextual factors substantially contribute to variation in sexual consent across experiences (Willis et al., 2021a). Therefore, in an exploratory manner, we provided a preliminary examination of how sexual consent is experienced for a diverse array of sexual behaviors (as well as diverse contexts within which sexual behavior might occur) that are frequently endorsed by people but not well represented in the empirical literature on sexual consent, which has instead focused on behaviors that align with traditional sexual scripts. Because gender has consistently been found to be associated with sexual consent (Jozkowski & Peterson, 2013; Willis, Hunt, et al., 2019), we also assessed the extent that gender differences were relevant across diverse sexual experiences in the present study.

## Sexual Consent

Informed by conceptual and empirical reviews, Willis and Jozkowski (2019, p. 1723) defined sexual consent as “one’s voluntary, sober, and conscious willingness to engage in a particular sexual behavior with a particular person within a particular context.” This definition maintains that sexual consent is an internal experience—one that is distinct from, but may be related to, sexual desire (Peterson & Muehlenhard, 2007). To assess the variety of feelings associated with an internal conceptualization of sexual consent, one research team asked participants to write about the feelings they associate with being willing to engage in sexual activity, specifically vaginal–penile sex (Jozkowski et al., 2014). These researchers consequently identified and validated five feelings related to internal consent: physical response, safety/comfort, arousal, agreement/want, and readiness. Thus, whether somebody is willing to engage in a particular behavior with a particular person within a particular context depends on a multidimensional process of internal feelings.

Because people cannot automatically know the feelings of others when they engage in partnered sexual activity, sexual consent should not only be conceptualized as an internal experience (Hickman & Muehlenhard, 1999). Rather, sexual partners externally communicate their consent (Beres, 2014; Muehlenhard et al., 2016). Active consent communication refers to any actions people do that indicate their consent and is diverse in practice; it can be verbal or nonverbal and explicit or implicit. People tend to rely on nonverbal consent cues more than verbal cues (Jozkowski et al., 2014; Muehlenhard et al., 2016). Examples of people’s self-reported nonverbal consent communication include moaning, positioning oneself to prepare for a sexual behavior, increasing physical contact, and making facial expressions. People also report communicating their sexual consent verbally—asking for sexual behavior directly, verbalizing sexual intent, or using seemingly benign phrases in a sexual tone (Hickman & Muehlenhard, 1999; Jozkowski et al., 2014). Active consent communication—even if it is implicit and nonverbal—is associated with higher levels of internal sexual consent (Willis, Blunt-Vinti, et al., 2019).

Even though internal consent feelings and active consent communication are related, their weak to moderate correlations suggest that these types of consent are separate and uniquely contribute to an overall conceptualization of sexual consent (Jozkowski et al., 2014; Walsh et al., 2019). Regarding the direction of the association between internal and external consent, Willis, Blunt-Vinti, et al. (2019) proposed a model whereby participants’ sexual consent feelings predicted their consent communication cues based on previous evidence that sexual cognitions tend to precede sexual behaviors.

### Sexual Consent and Type of Sexual Behavior

People do not experience or communicate their willingness to engage in sexual activity the same way across contexts. For example, sexual consent tends to vary by type of sexual behavior. Regarding sexual consent feelings, participants in a daily diary study reported greater internal consent for sexual events that involved vaginal–penile sex than those that only involved passionate kissing, genital touching, or oral sex (Willis et al., 2021a). Similarly, Marcantonio et al. (2018) found that ratings of physical response, safety/comfort, arousal, agreement/want, and readiness were higher for people’s most recent vaginal–penile sex than for their most recent experiences of genital touching or oral sex.

External sexual consent also varies by type of behavior. One of the first empirical studies on types of consent communication assessed a relatively broad range of intimate and sexual behaviors: hugging, kissing, breast touching, genital touching, oral sex, orgasm, vaginal–penile sex, and anal sex (Hall, 1998). Descriptive statistics suggested that people were more likely to actively communicate their willingness using either verbal or nonverbal cues to behaviors such as oral, vaginal–penile, or anal sex compared with behaviors like hugging or touching breasts. A more recent study similarly found that explicit verbal cues were reported with significantly increasing frequency for the following sexual behaviors: intimate touching (22.0%), oral sex (43.5%), vaginal–penile sex (57.4%), and anal sex (80.1%; Willis, Hunt, et al., 2019). In addition to type of sexual behavior, direction matters. In that same study, active consent communication seemed to be more prominent for performing oral sex than receiving oral sex.

In sum, people’s experiences of both internal and external sexual consent can depend on the type of sexual behavior they are engaging in. Research to date has only examined sexual consent as it relates to a handful of sexual behaviors—primarily those that fit within general conceptualizations of having had “sex” (Barnett et al., 2017; Sanders et al., 2010). However, people are much more diverse in their sexuality (Herbenick et al., 2017). Additional contexts and broader conceptualizations of sexual behavior must be considered to better understand people’s lived experiences of sexual consent. Further, gender differences may persist across types of sexual behavior and should be considered.

### Sexual Consent and Gender

According to the traditional sexual script, people who identify as women are more likely to be the gatekeeper in a given encounter and thus accept or rebuff a male initiator’s attempt for sex (Curtis & Burnett, 2017; Jozkowski & Peterson, 2013). Based on these stereotypically gendered roles, both women and men tend to describe sexual consent as something men get from women (Righi et al., 2019). Because women are reinforced as gatekeepers and subsequently experience inhibited sexual agency, they tend to communicate their willingness to engage in sexual activity relatively less directly than men (Curtis & Burnett, 2017; Jozkowski & Peterson, 2013). Evidencing this, Willis, Hunt, et al. (2019) found that men were more likely than women to use explicit verbal cues relative to implicit nonverbal cues.

As for internal sexual consent, the existing literature is limited and mixed but generally indicates that gender differences may depend on the feeling in question. For example, Jozkowski et al. (2014) found that women reported lower levels of arousal and higher levels of safety and comfort than men. A different study found that women scored higher on physical response (Walsh, Honickman, et al., 2019). Other areas of literature provide some insight with their comparisons of women and men on individual aspects of internal consent. There is evidence that men report higher levels of physical response (Milhausen et al., 2010), arousal (Chivers, 2005), and want (Hatfield et al., 1989). Thus, the general impression of extant research is that women report experiencing diminished levels of internal sexual consent compared with men, but more research is warranted.

Gender may even moderate associations between sexual consent and type of sexual behavior. For instance, Hall (1998) found that men were more likely than women to communicate their consent either verbally or nonverbally for genital touching and breast stimulation; however, consent for oral sex or vaginal–penile sex was communicated similarly across gender. Overall, gender likely remains relevant even when considering how people experience and communicate sexual consent for diverse sexual behaviors.

### Nonconsensual Sexual Experiences

While sexual consent is an important construct to examine in its own right, the absence of sexual consent has a more robust empirical history regarding its antecedents and consequences. Experiencing nonconsensual sexual activity has been associated with detrimental effects to victims’ well-being—sexually, mentally, emotionally, and physically (e.g., Campbell et al., 2009; Gidycz et al., 2008). Like previous research on sexual consent, the extant literature on nonconsensual sex has focused on a small selection of behaviors. For example, the Sexual Experiences Survey (i.e., a widely used measure of nonconsensual sexual activity) asks about sexual behaviors such as kissing, fondling, genital touching, oral sex, vaginal sex, anal sex (Koss et al., 2007). Again, such narrow conceptualizations do not adequately encompass people’s sexual diversity across contexts. Extending research on nonconsensual sexual activity to include experiences that might not legally classify as sexual assault (e.g., sexual touching against somebody’s will) or rape (e.g., forced penetration) is important because consent is pertinent and should be emphasized for all sexual behaviors—disregarding whether certain types of physical contact are involved.

Across behaviors, women are at a significantly greater risk of experiencing nonconsensual sexual activity than men. In the United States, 43.9% of women and 23.4% of men reported having experienced sexual violence during their lifetime (Breiding, 2014). For these reasons, we extended our examination of sexual consent regarding diverse sexual behaviors to include the absence of consent.

## Present Study

Despite evidence supporting widespread sexual diversity (Herbenick et al., 2017), extant literature on sexual consent has been limited to sexual behaviors that may be considered “typical” based on people’s conceptualizations of sex. Because sexual consent varies so much from one context to the next, sexual consent feelings and communication regarding behaviors like vaginal–penile sex may not generalize to more diverse—or even stigmatized—sexual behaviors or contexts. In the present study, we examined people’s willingness—and active communication of that willingness—to engage in a broader array of sexual experiences than any other research has done to our knowledge. We did not make behavior-specific hypotheses regarding internal or external sexual consent.

Given the consistent effects of gender throughout the literature on sexual consent (Jozkowski et al., 2014; Willis, Hunt, et al., 2019), we predicted that women and men would differ in their internal and external sexual consent to various types of sexual behaviors. Specifically, we expected women to report lower levels of sexual consent feelings and lower levels of active consent communication.

Finally, we sought to provide preliminary evidence regarding people’s experiences of diverse and potentially stigmatized sexual behaviors that occurred against their will or without their consent. Because women disproportionately experience nonconsensual sexual activity (Breiding, 2014), we predicted that this gender disparity would persist for nonconsensual experiences with various sexual behaviors.

## Method

### Participants

To determine which sexual behaviors to include in the present study, we first conducted a pilot study (*N* = 218; 60.9% women). For the full study, we ensured that participants were more evenly distributed regarding gender (*N* = 658; 50.5% women). Table 1 presents the sociodemographic characteristics for the full sample by gender.

**Table 1.** Sociodemographic Characteristics.

Variable	Women(*n* = 332)	Men(*n* = 326)
Age		
*M* (*SD*)	32.3 (11.6)	32.11 (11.8)
Country of residence		
United Kingdom	169 (50.9%)	162 (49.7%)
United States	163 (49.1)	164 (50.3)
Race/ethnicity		
White	253 (76.2)	256 (78.5)
Black	25 (7.5)	15 (4.6)
Asian	23 (6.9)	24 (7.4)
Hispanic	12 (3.6)	15 (4.6)
Multiracial/other	19 (5.7)	16 (4.9)
Education level		
A-Levels/high school or less	72 (21.7)	76 (23.3)
Some university but no degree	81 (24.4)	77 (23.6)
Bachelor’s degree	111 (33.4)	113 (34.7)
Master’s degree	57 (17.2)	42 (12.9)
Doctoral/professional degree	11 (3.3)	18 (5.6)
Student status		
Currently a student	86 (25.9)	81 (24.8)
Not a student	246 (74.1)	245 (75.2)
Household income		
Less than £/$20,000	71 (21.4)	63 (19.4)
£/$20,000 to £/$39,999	82 (24.6)	74 (22.7)
£/$40,000 to £/$59,999	74 (22.3)	70 (21.5)
£/$60,000 to £/$79,999	44 (13.2)	47 (14.4)
£/$80,000 to £/$99,999	22 (6.6)	27 (8.3)
£/$100,000 or more	39 (11.7)	45 (13.8)
Sexual orientation^1^		
Heterosexual	255 (76.8)	286 (87.7)
Homosexual	9 (2.7)	17 (5.2)
Bisexual	55 (16.6)	18 (5.5)
Unsure/other	13 (3.9)	5 (1.5)
Current sexual partners^1^		
0 partners	83 (25.0)	101 (31.0)
1 partner	238 (71.7)	205 (62.9)
2+ partners	11 (3.3)	20 (6.2)

*Notes.* Gender was significantly associated with sexual orientation, *χ*^2^(1) = 26.49, *p* < .001, *φ*_C_ = .20, and number of sexual partners, *χ*^2^(1) = 6.78, *p* = .034, *φ*_C_ = .10. No other sociodemographic variables significantly differed by gender.

### Procedure

Participants for the pilot study and full study were recruited to complete an online cross-sectional survey via Prolific Academic, which is a large-scale data collection service based in the United Kingdom.^1^ The text advertising the study read, “In this study, we will ask you about your previous sexual experiences.” Eligibility criteria included being at least 18 years old and a resident in the United Kingdom or the United States, and quotas were in place to obtain a sample that was approximately evenly distributed regarding proportions of women and men. The survey took about 10-15 minutes to complete (5-8 minutes for the pilot study). Participants in the full study received £2.92 GBP or US$3.50 for their contribution. The procedure for this study was approved by the university’s research ethics committee.

### Measures

*Types of Sexual Behavior.* Based on previous research, we drafted an initial list of 30 sexual behaviors that people engage in (see Appendix). Most behaviors and contexts were adapted from Herbenick et al.’s (2017) study on sexual diversity; we added substance-involved sexual behaviors due to the importance of considering alcohol and drugs when assessing sexual consent (e.g., Willis et al., 2021b). We conducted a pilot study that asked participants which of these behaviors they had engaged in. Participants also had the opportunity to describe other types of sexual behaviors they have experienced in an open-ended format. We used data from the pilot study to amend our list of sexual behaviors and reduce the number to 20 with the intent of decreasing participant fatigue during the full study.

Based on low endorsement, we removed sexual behaviors that explicitly involved strangers, transactional sex, or power differences. Further, we used open-ended pilot data to clarify some sexual behaviors. For example, even though group sex was not frequently endorsed by pilot participants in the closed-ended items, several mentioned “threesomes” in their open-ended responses for other types of sexual behaviors they had engaged in. Therefore, we retained this behavior for the present study but added clarification. Finally, we collapsed some behaviors that were separate in the pilot study: (e.g., phone sex and video sex became “phone or video sex,” anal stimulation and anal penetration became “anal stimulation or penetration”).

The final list of 20 sexual behaviors included in the full study captured various body parts (e.g., oral-genital contact, anal stimulation), combinations of people (e.g., two people of the same gender, group sex), substance use (e.g., alcohol, other drugs), enhancers (e.g., sex toys, role playing), or technology (e.g., sexting, phone/video sex). Participants selected all of the sexual behaviors that they had experienced at least some point in their life. Participants could also select an item that read “I have not experienced any of these behaviors.” See Supplemental Material for the exact phrases we used to describe sexual behaviors.

*Internal and External Sexual Consent.* For each type of sexual behavior participants endorsed, they were asked to report their internal and external sexual consent during their most recent experiences with those behaviors. Because participants completed measures of sexual consent for each of the 20 types of sexual behaviors they had experienced, we did not administer the complete 25-item Internal Consent Scale or 18-item External Consent Scale (Jozkowski et al., 2014). Rather, we used shorter measures that were developed and validated to reduce participant fatigue when assessing sexual consent several times (Willis et al., 2021c).

For internal consent, five items reflected the factors of the Internal Consent Scale: physical response, safety/comfort, arousal, agreement/want, and readiness (Jozkowski et al., 2014). For external consent, four items assessed the core aspects of active consent communication: explicit, implicit, verbal, and nonverbal (Willis, Blunt-Vinti, et al., 2019). These 9 items were all rated on a 4-point Likert-type scale (*Strongly disagree* to *Strongly agree*).

We created composite scores by averaging items scores that mapped onto each construct: internal sexual consent (sample *α* = .91) and external sexual consent (sample *α* = .76). Higher scores indicate greater levels of sexual consent feelings or greater use of active consent communication, respectively.

*Nonconsensual Sexual Experiences.* Finally, we asked participants whether they had ever experienced any of the same 20 sexual behaviors “without their consent or against their will.” We manipulated the presence of force in the items; participants randomly responded to either a set of behaviors that included force (e.g., “Somebody has forced me to have vaginal–penile sex against my will”) or a set that did not (e.g., “I have had vaginal–penile sex against my will”). Participants checked all that applied and had the option to indicate that they had never experienced any of the sexual behaviors against their will.

### Analysis

To provide an exploratory assessment of people’s willingness to engage in diverse sexual behaviors, we calculated descriptive statistics for internal and external sexual consent by type of sexual behavior. Using independent samples *t* tests, we compared ratings of sexual consent by gender for each sexual behavior. Regarding gender differences in experiences of nonconsensual sexual activity, we conducted chi-squared tests of independence.

All tests of significance were conducted at an *α* level of .05. We reported Cohen’s *d* as an effect size for each *t* test and Cremer’s V (φ_C_) for each of the chi-squared tests. According to Cohen (1988), a *d*-value of .2 indicates a small effect size, .5 medium, and .8 large; corresponding values for φ_C_ are .1, .3, and .5, respectively. All data preparation and analyses were conducted using SPSS 26.

**Table 2.** Descriptive Statistics for Sexual Consent Variables (N = 658).

Type of Behavior	Lifetime Prevalence *n* (%)	Internal Consent *M* (*SD*)	External Consent *M* (*SD*)	Nonconsensual Experiences^1^ *n* (%)
Vaginal–penile sex	569 (86.5)	3.61 (.53)	3.31 (.58)	86 (13.1)
Sex toy	351 (53.3)	3.58 (.54)	3.36 (.61)	12 (1.8)
BDSM	180 (27.4)	3.56 (.55)	3.37 (.62)	8 (1.2)
Different gender	528 (80.2)	3.56 (.62)	3.29 (.66)	62 (9.4)
Oral sex (receive)	590 (89.7)	3.55 (.60)	3.23 (.63)	60 (9.1)
High sex	204 (31.0)	3.54 (.62)	3.27 (.64)	17 (2.6)
Drunk sex	435 (66.1)	3.49 (.58)	3.25 (.63)	51 (7.8)
Oral sex (give)	581 (88.3)	3.49 (.56)	3.24 (.57)	78 (11.9)
Role play	219 (33.3)	3.48 (.58)	3.29 (.61)	9 (1.4)
Online dating	236 (35.9)	3.48 (.57)	3.24 (.65)	18 (2.7)
Watched porn	317 (48.2)	3.41 (.59)	3.16 (.65)	9 (1.4)
Sex tape	138 (21.0)	3.41 (.66)	3.15 (.68)	9 (1.4)
Same gender	149 (22.6)	3.41 (.71)	3.29 (.65)	16 (2.4)
Phone/video sex	263 (40.0)	3.40 (.60)	3.19 (.61)	14 (2.1)
Group sex	120 (18.2)	3.39 (.63)	3.24 (.66)	15 (2.3)
Anal sex (give)	256 (38.9)	3.38 (.65)	3.17 (.66)	17 (2.6)
Public sex	320 (48.6)	3.37 (.61)	3.22 (.65)	25 (3.8)
Sext (sent)	346 (52.6)	3.28 (.62)	3.06 (.70)	17 (2.6)
Sext (receive)	400 (60.8)	3.22 (.83)	2.94 (.84)	85 (12.9)
Anal sex (receive)	250 (38.0)	3.03 (.81)	2.95 (.76)	53 (8.1)
*Total*	632 (96.0)	3.45 (.63)	3.21 (.66)	204 (31.0)

*Notes*. Sexual behaviors are listed in descending order regarding average internal consent scores.

^1^Participants were randomly assigned to a condition that asked about their sexual experiences that were either “against their will” or “forced against their will.”

**Table 3.** Lifetime Prevalence of Sexual Experiences by Gender.

Type of Behavior	Women *n* (%)	Men *n* (%)	*χ*^2^-statistic	*p*-value	Cramer’s V
Oral sex (give)	293 (88.3)	273 (83.7)	2.78	.095	.07
Oral sex (receive)	291 (87.7)	287 (88.0)	0.02	.880	.01
Vaginal–penile	293 (88.3)	265 (81.3)	6.19*	.013	.10
Anal sex (give)	93 (28.0)	155 (47.5)	26.73***	<.001	.20
Anal sex (receive)	175 (52.7)	66 (20.2)	74.69***	<.001	.34
Different gender	273 (82.2)	248 (76.1)	3.78	.052	.08
Same gender	87 (26.2)	55 (16.9)	8.47**	.004	.11
Group sex	57 (17.2)	58 (17.8)	0.04	.833	.01
Online dating	113 (34.0)	116 (35.6)	0.17	.677	.02
Drunk sex	229 (69.0)	199 (61.0)	4.55*	.033	.08
High sex	104 (31.3)	93 (28.5)	0.61	.433	.03
Public sex	178 (53.6)	139 (42.6)	7.94**	.005	.11
Sex toy	192 (57.8)	150 (46.0)	9.21**	.002	.12
Role play	111 (33.4)	101 (31.0)	0.45	.501	.03
BDSM	99 (29.8)	73 (22.4)	4.70*	.030	.08
Sext (sent)	181 (54.5)	156 (47.9)	2.92	.087	.07
Sext (receive)	194 (58.4)	197 (60.4)	0.27	.602	.02
Phone/video sex	128 (38.6)	128 (39.3)	0.03	.852	.01
Watched porn	169 (50.9)	143 (43.9)	3.27	.071	.07
Sex tape	66 (19.9)	71 (21.8)	0.36	.548	.02

*Note.* ^1^This percentage uses the number of participants who reported engaging in each type of sexual behavior as the denominator—rather than the total number of participants.

**p < .01. ***p < .001.

## Results

### Descriptive Statistics

Overall, 632 (96.0%) of participants had engaged in at least one of the sexual behaviors listed. Of those, participants on average reported experiences with 10 of the 20 types of sexual behavior listed (*SD* = 4.6), ranging from 1 to 20. The most endorsed sexual behaviors were receiving oral sex (89.7%), giving oral sex (86.5%), and vaginal–penile sex (86.5%). The least endorsed sexual behaviors were group sex (18.2%), making a sex tape (21.0%), and having sex with somebody of the same gender (22.6%). Table 2 presents prevalence rates for all other behaviors; gender differences in lifetime prevalence rates of each sexual behavior appear in Table 3.

The average event-level internal sexual consent score across all behaviors was 3.45 (*SD* = .63). Sexual behaviors with the greatest levels of internal consent were vaginal–penile sex (*M* = 3.61), using a sex toy (*M* = 3.58), and engaging in bondage-dominance/sadism-masochism (BDSM; *M* = 3.56). Sexual behaviors with the lowest levels of internal consent were receiving anal stimulation (*M* = 3.03), receiving a sexually explicit photo or video (i.e., sext; *M* = 3.22), and sending a sext (*M* = 3.28). Table 2 presents average internal sexual consent scores for all other behaviors.

There was a pattern that behaviors with greater levels of internal sexual consent were associated with greater levels of active consent communication. Indeed, across all behaviors assessed internal and external sexual consent were significantly and positively correlated, *r* = .67, *p* < .001. Further, the three sexual behaviors with the greatest levels of external consent were the same as the top three for internal consent: vaginal–penile sex (*M* = 3.31), using a sex toy (*M* = 3.36), and engaging in BDSM (*M* = 3.37). This was true for the bottom three behaviors regarding external consent: receiving anal stimulation (*M* = 2.95), receiving a sexually explicit photo or video (i.e., sext; *M* = 2.94), and sending a sext (*M* = 3.06). Table 2 presents average active consent communication scores for all other behaviors.

Almost a third of participants (*n* = 204; 31.0%) had experienced at least one of the listed sexual behaviors against their will. Of those, participants on average reported nonconsensual experiences with 3.1 of the 20 types of sexual behavior listed (*SD* = 2.1), ranging from 1 to 11. The sexual behaviors participants were most likely to have experienced against their will were vaginal–penile sex (13.1%), receiving a sext (12.9%), and giving oral sex (11.9%). Table 2 presents prevalence rates of nonconsensual sexual experiences.

### Sexual Consent by Gender

Women reported significantly lower sexual consent feelings than men for 12 of 20 sexual behaviors; there were no other significant differences. The significant gender differences with the largest effect sizes were receiving a sext (*t* = 8.34, *p* < .001, Cohen’s *d* = .84), making a sex tape (*t* = 3.80, *p* < .001, Cohen’s *d* = .66), and giving anal stimulation (*t* = 3.87, *p* < .001, Cohen’s *d* = .84). Table 4 presents test statistics and effects size regarding internal sexual consent for all other behaviors.

**Table 4.** Internal Sexual Consent by Gender.

Type of Behavior	Women		Men		*t*-statistic	*p*-value	Cohen’s *d*
	*n*	*M* (*SD*)	*n*	*M* (*SD*)			
Oral sex (give)	293	3.36 (.61)	274	3.63 (.43)	6.12***	<.001	.51
Oral sex (receive)	291	3.42 (.66)	288	3.71 (.45)	6.01***	<.001	.50
Vaginal–penile	293	3.50 (.59)	265	3.72 (.42)	5.12***	<.001	.43
Anal sex (give)	93	3.16 (.78)	155	3.51 (.51)	3.87***	<.001	.53
Anal sex (receive)	175	2.97 (.81)	67	3.19 (.78)	1.96	.051	.28
Different gender	271	3.47 (.67)	248	3.67 (.54)	3.84***	<.001	.34
Same gender	86	3.49 (.59)	55	3.32 (.82)	-1.35	.180	.24
Group sex	57	3.24 (.71)	59	3.52 (.54)	2.40*	.018	.44
Online dating	113	3.39 (.63)	116	3.57 (.49)	2.29*	.020	.30
Drunk sex	229	3.50 (.58)	199	3.50 (.53)	0.00	.999	.00
High sex	104	3.45 (.75)	94	3.62 (.45)	1.95	.053	.27
Public sex	177	3.25 (.67)	139	3.51 (.47)	4.04***	<.001	.45
Sex toy	192	3.56 (.53)	150	3.59 (.56)	0.46	.644	.05
Role play	111	3.44 (.62)	101	3.52 (.53)	1.03	.307	.14
BDSM	98	3.51 (.59)	73	3.62 (.50)	1.29	.200	.20
Sext (sent)	181	3.13 (.65)	156	3.45 (.54)	4.93***	<.001	.54
Sext (receive)	194	2.90 (.88)	196	3.54 (.61)	8.34***	<.001	.84
Phone/video sex	128	3.26 (.67)	128	3.54 (.49)	3.78***	<.001	.47
Watched porn	168	3.37 (.63)	143	3.46 (.55)	1.34	.181	.15
Sex tape	65	3.18 (.74)	71	3.60 (.50)	3.80***	<.001	.66

*Note.* *p < .05. ***p < .001.

**Table 5.** External Sexual Consent by Gender.

Type of Behavior	Women		Men		*t*-statistic	*p*-value	Cohen’s *d*
	*n*	*M* (*SD*)	*n*	*M* (*SD*)			
Oral sex (give)	293	3.17 (.60)	274	3.32 (.52)	3.17**	.002	.27
Oral sex (receive)	291	3.16 (.67)	288	3.31 (.57)	2.93**	.004	.24
Vaginal–penile	293	3.26 (.62)	265	3.36 (.54)	2.09*	.037	.18
Anal sex (give)	93	3.05 (.74)	155	3.23 (.60)	2.10*	.036	.27
Anal sex (receive)	175	2.89 (.75)	67	3.11 (.74)	2.04*	.042	.29
Different gender	271	3.25 (.69)	248	3.34 (.62)	1.56	.119	.14
Same gender	86	3.33 (.59)	55	3.21 (.76)	-1.06	.291	.18
Group sex	57	3.11 (.67)	59	3.33 (.65)	1.76	.081	.33
Online dating	113	3.23 (.65)	116	3.25 (.64)	0.16	.876	.02
Drunk sex	229	3.29 (.64)	199	3.22 (.58)	-1.17	.243	.11
High sex	104	3.22 (.64)	94	3.32 (.64)	1.13	.261	.16
Public sex	177	3.15 (.67)	139	3.31 (.61)	2.17*	.031	.25
Sex toy	192	3.37 (.59)	150	3.33 (.63)	-0.59	.554	.06
Role play	111	3.31 (.61)	101	3.26 (.60)	-0.64	.525	.09
BDSM	98	3.38 (.64)	73	3.34 (.59)	-0.44	.663	.07
Sext (sent)	181	3.01 (.71)	156	3.12 (.68)	1.46	.146	.16
Sext (receive)	194	2.77 (.89)	196	3.12 (.72)	4.26***	<.001	.43
Phone/video sex	128	3.17 (.63)	128	3.20 (.60)	0.36	.722	.04
Watched porn	168	3.17 (.67)	143	3.12 (.63)	-0.69	.493	.08
Sex tape	65	3.08 (.67)	71	3.20 (.68)	0.97	.334	.17

*Note.* *p < .05. **p < .01. ***p < .001.

**Table 6.** Nonconsensual Sexual Experiences by Gender.

Type of Behavior	Women		Men		*χ*^2^-statistic	*p*-value	Cramer’s V
	*n* (%)	% within behavior^1^	*n* (%)	% within behavior^1^			
Oral sex (give)	55 (16.6)	18.8	20 (6.1)	7.3	18.07***	<.001	.17
Oral sex (receive)	35 (10.5)	12.0	23 (7.0)	8.0	2.60	.107	.06
Vaginal–penile	70 (21.1)	23.9	12 (3.6)	4.5	46.24***	<.001	.26
Anal sex (give)	7 (2.1)	7.5	10 (3.0)	6.5	0.57	.450	.03
Anal sex (receive)	38 (11.4)	21.7	14 (4.3)	20.9	11.79***	<.001	.13
Different gender	51 (15.4)	18.9	8 (2.4)	3.2	33.98***	<.001	.23
Same gender	4 (1.2)	4.7	11 (3.3)	20.0	3.41	.065	.07
Group sex	8 (2.4)	14.0	7 (2.1)	11.9	0.06	.808	.01
Online dating	9 (2.7)	8.0	9 (2.7)	7.8	0.00	.984	.00
Drunk sex	36 (10.8)	15.7	13 (4.0)	6.5	11.44***	<.001	.13
High sex	9 (2.7)	8.7	8 (2.4)	8.5	0.05	.821	.01
Public sex	18 (5.4)	10.2	5 (1.5)	3.6	7.49**	.006	.11
Sex toy	7 (2.1)	3.6	5 (1.5)	3.3	0.32	.571	.02
Role play	2 (0.6)	1.8	7 (2.1)	6.9	2.86	.091	.07
BDSM	3 (0.9)	3.1	4 (1.2)	5.5	0.15	.695	.02
Sext (sent)	10 (3.0)	5.5	7 (2.1)	4.5	0.52	.473	.03
Sext (receive)	59 (17.8)	30.4	21 (6.4)	10.7	20.15***	<.001	.17
Phone/video sex	8 (2.4)	6.3	5 (1.5)	3.9	0.68	.410	.03
Watched porn	2 (0.6)	1.2	7 (2.1)	4.9	2.86	.091	.07
Sex tape	7 (2.1)	10.8	2 (0.6)	2.8	2.77	.096	.06

*Notes.* ^1^This percentage uses the number of participants who reported engaging in each type of sexual behavior as the denominator—rather than the total number of participants.

**p < .01. ***p < .001.

Women reported significantly lower active consent communication than men for 7 of 20 sexual behaviors; there were no other significant differences. The significant gender differences with the largest effect sizes were receiving a sext (*t* = 4.26, *p* < .001, Cohen’s *d* = .43), receiving anal stimulation (*t* = 2.04, *p* = .042, Cohen’s *d* = .29), and giving anal stimulation (*t* = 2.10, *p* = .036, Cohen’s *d* = .27). Table 5 presents test statistics and effect sizes regarding external sexual consent for all other behaviors.

### Nonconsensual Sexual Experiences by Gender

We assessed gender differences in overall victimization rates based on experimental condition. For participants who were only asked about experiences that happened “against their will,” 47.9% of women reported at least one nonconsensual experience with one of the sexual behaviors assessed compared with 22.3% of men, χ^2^(1) = 23.80, *p* < .001, φ_C_ = .27.

For those who were asked about experiences that were “forced” and “against their will,” 38.3% of women reported at least one nonconsensual experience with one of the sexual behaviors assessed compared with 16.0% of men, χ^2^(1) = 20.82, *p* < .001, φ_C_ = .25.

We collapsed conditions when comparing women and men on nonconsensual experiences with individual sexual behaviors. Women were more likely than men to report nonconsensual experiences with 7 of 20 sexual behaviors; there were no other significant differences. The significant gender differences with the largest effect sizes were vaginal–penile sex (χ^2^ = 46.24, *p* < .001, φ_C_ = .26), having sex with a different gender (χ^2^ = 33.98, *p* < .001, φ_C_ = .23), and receiving a sext (χ^2^ = 20.15, *p* < .001, φ_C_ = .17). For example, 21.1% of women reported having experienced nonconsensual vaginal–penile sex, while only 3.6% of men reported the same. Table 6 presents proportions, test statistics, and effect sizes regarding nonconsensual sexual experiences for all other behaviors; this table also includes proportions that were based on the number of participants who reported ever engaging in each sexual behavior.

## Discussion

Extant research on sexual consent has consistently focused on a few “typical” sexual behaviors, such as oral sex and vaginal–penile sex. While our study suggested that these sexual behaviors may be the most common, there are many other widely endorsed sexual experiences that had not previously been evaluated regarding sexual consent. Corroborating previous studies (e.g., Marcantonio et al., 2018; Willis, Hunt, et al., 2019), we found that people’s internal and external sexual consent varied across types of sexual behaviors and contexts.

The behavior with the highest levels of internal sexual consent was vaginal–penile sex. This finding aligns with those from other studies (Marcantonio et al., 2018; Willis et al., 2021a). Because vaginal–penile intercourse is considered by most to be synonymous with “sex” (Sanders et al., 2010), this sexual behavior is likely the least stigmatized or tabooed—which may be related with greater comfort engaging in vaginal–penile sex as well as greater comfort actively communicating willingness to engage in this behavior.

Other sexual behaviors associated with elevated levels of internal sexual consent may align with norms that are supportive of active consent communication. For example, engaging in BDSM and using sex toys were the behaviors with the highest levels of external consent and, perhaps consequently, were also at the top of the list regarding internal consent. Indeed, BDSM subcultures centralize sexual consent and open communication as core components of that behavior (Fanghanel, 2020). That sexual consent feelings were positively associated with active consent communication in the present study supports previous findings (Jozkowski et al., 2014; Willis, Blunt-Vinti, et al., 2019).

Another two behaviors worth noting were above the median regarding internal sexual consent are drunk sex and high sex. Despite the potential impairing effects of alcohol and other drugs on people’s capacity to consent, substance-involved sexual activity is common, and people frequently label their substance-involved sexual activity as consensual (Herbenick et al., 2019; Jozkowski & Wiersma, 2015). Indeed, substance use can even be perceived as part of the sexual consent communication process (King et al., 2021); by drinking alcohol or smoking cannabis with somebody else, a person might think they are actively communicating their willingness to engage in sexual activity. However, substance use remains a notable risk factor for nonconsensual sexual activity given that alcohol is involved in about half of sexual assaults (Abbey et al., 2001) and men are more likely to use alcohol or drugs to facilitate sexual assault than other strategies (Gidycz et al., 2011). Further, recently published data suggested that level of impairment should be emphasized when considering consent to substance-involved sexual activity; of note, participants in that study reported diminished levels of internal consent feelings during sexual events that involved either greater alcohol consumption or the combined use of alcohol and cannabis (Willis et al., 2021b). Future work on how people differentiate consensual versus nonconsensual substance-involved sexual activity is warranted.

Sexual behaviors on the lower end of the internal consent spectrum may have norms less supportive of active consent communication. For example, our finding that anal sex demonstrated the lowest levels of internal and external sexual consent reflects previous evidence that this behavior may be associated with sexual coercion and decreased sexual agency (Fahs & Gonzalez, 2014; Maynard et al., 2009). Sexting was another behavior associated with diminished sexual consent. Because this behavior takes place in virtual settings and does not involve physical contact, people may not perceive the importance of communicating willingness to engage in sexting or other behaviors that are considered less intimate (Humphreys, 2007). However, given the potential effect sexting can have on people’s sexual and mental health (Mori et al., 2019), sexual consent remains as important for this and other virtual sexual behaviors as it does for physical sexual behaviors.

### Sexual Consent and Gender

We found that women tended to report lower levels of internal sexual consent than men. Sexual behaviors for which women and men reported similarly experiencing willingness included using a sex toy, engaging in role play, engaging in BDSM, and having sex with somebody of the same gender. Each of these sexual behaviors may diminish stereotypical gender roles and permit women spaces to actively communicate their willingness—consequently increasing their sexual agency, which can lead to better, healthier, and more consensual sexual experiences (Mark & Vowels, 2020).

Unfortunately, the traditional sexual script does not afford women much sexual agency and positions them as the gatekeepers of sexual activity (Curtis & Burnett, 2017; Jozkowski & Peterson, 2013). That men are traditionally the initiators of sexual activity may be the reason that they had greater levels of internal sexual consent for most behaviors that do not promote egalitarian sexual agency. Further reflecting their socially prescribed sexually agentic roles, men also reported engaging in active consent communication to a greater extent than women; however, these gender differences were much smaller than those for internal consent.

### Nonconsensual Sexual Experiences

Women’s diminished feelings of consent during their most recent sexual encounters may reflect their greater risk for experiencing nonconsensual sexual activity. Indeed, we found that 43% of women and 19% of men reported a nonconsensual experience with at least one of the behaviors listed. Of those who had been sexually victimized, the average person had nonconsensual experiences with three of the sexual behaviors. Such polyvictimization may increase risk for negative outcomes like depressive symptoms or posttraumatic stress (Sabena & Straus, 2008).

Participants were less likely to report nonconsensual experiences if they were prompted to think of “forced” encounters. Researchers should recognize that sexual assault can exist with or without force and that prevalence rates will likely vary based on item wording (Rueff & Gross, 2017; Strang et al., 2013). Even though some of the nonconsensual experiences assessed in the present study would not qualify as sexual assault or rape (e.g., nonconsensual sexting), they can still have negative effects on people’s health and well-being (Mori et al., 2019). Indeed, the sexual health and mental health outcomes victims experience vary by the type of nonconsensual behavior (Pinsky et al., 2017).

## Implications

A key component of preventing nonconsensual sexual activity is understanding and promoting healthy sexual consent practices. Our findings have several implications for such initiatives. Specifically, sexual diversity should be embraced, sexual agency should be emphasized, and active consent communication should be encouraged.

First, there are growing concerns that sexual consent education programs do not adequately reflect contemporary people’s sexually diverse lived experiences (Herbenick et al., 2017). Relying solely on teaching examples that depict “typical” sexual behaviors is a disservice to the nuances of sexual consent, which warrant empirically informed discussions. Instead, people should be taught that consent matters for all sexual behaviors—even those that do not involve physical contact. For example, curricula should acknowledge that the process of communicating sexual consent—and even sexual behavior itself—increasingly takes places in virtual spaces. As such, sexual consent education programs should address navigating sexual consent for behaviors such as sexting, online dating, and phone/video sex. Other examples of widely endorsed sexual behaviors that should not be ignored include same-gender sex, group sex, substance-involved sex, and the use of sexual enhancers.

Second, initiatives aimed at promoting sexual consent and preventing nonconsensual sexual activity should emphasize sexual agency. Women’s stereotypically gendered role in the sexual consent process devalues their rightful position as an equal player in sexual encounters. Thus, the social institution of gender acts as a barrier to positive sexual consent practices (Willis & Jozkowski, 2018). Promoting egalitarian sexual consent practices may help diminish the detrimental effect of traditional gender roles. Supporting this recommendation, we found that behaviors that may lend themselves to more active participation and respect from all people involved in a sexual encounter (e.g., using sex toys, engaging in BDSM) had relatively high levels of internal and external sexual consent.

Finally, active consent communication should be encouraged. Many consent education programs currently prioritize this approach (Curtis & Burnett, 2017). While we would not recommend that communication be a single-pronged approach to promoting health sexual consent or preventing sexual violence, our findings corroborated previous evidence that active consent communication is associated with greater levels of willingness to engage in a sexual behavior (Jozkowski, et al., 2014; Willis, Blunt-Vinti, et al., 2019). Because people may feel less comfortable openly communicating their willingness to engage in sexual behaviors that are stigmatized or tabooed, embracing positive sexuality and sexual diversity may be an effective way to foster active consent communication.

## Strengths and Limitations

The composition of our sample represents a strength and a limitation of the present study. Most previous research on sexual consent has relied on data from university students (Willis, Blunt-Vinti, et al., 2019); however, we collected data from participants who were mostly not students at the time of the study and were more diverse regarding age. Further, many studies on gender differences in sexual consent have relied on samples that were disproportionately female (Jozkowski et al., 2014; Willis, Hunt, et al., 2019), which can bias parameter estimates. To test more valid comparisons, we collected a sample that had about as many women as men. Yet our sample remained limited regarding its representation of other sociodemographic characteristics (e.g., sexual orientation, race/ethnicity). Future studies on sexual consent should collect samples that are more representative of diverse sexual identities, which would help inform how sexual consent is experienced and communicated during sexual encounters that may not be as subjected to traditional gender roles and heterosexism.

Another strength of our study was that its design reduced potential participant fatigue and thus may have better accommodated diverse individual differences regarding attentional capacity. Specifically, we conducted a pilot study to shorten our list of sexual behaviors and used measures that were developed and validated as brief assessments of sexual consent (Willis et al., 2021c). Given the novel and exploratory nature of the present study, a retrospective cross-sectional survey was an appropriate use of resources to provide preliminary data on people’s experiences of sexual consent to a diverse array of sexual behaviors. But retrospective measures of sexual behaviors are subjected to memory biases (Willis & Jozkowski, 2018), and this design is unable to provide insight regarding day-to-day variability, which is highly relevant for sexual consent (Willis et al., 2021a). Future work should be designed to build on the present study by using approaches like experience sampling methodology to assess people’s daily sexual diversity.

Although our study acknowledged people’s sexual diversity by emphasizing breadth of sexual behaviors, our analyses were limited in the depth of information they could provide regarding the extent that sexual consent varies at the intersection of multiple contexts. For example, a person’s willingness to engage in sexual activity may be particularly important to consider when they are engaging in drunk sex with somebody of the same gender that they met online—yet, in the present study, we only assessed each of these three contexts on their own. Indeed, we could have assessed the potential moderating effects of notable interpersonal contexts (e.g., gender of partner, relationship status with partner) across each type of sexual behavior to provide a more complete account the contextual variability of sexual consent; however, asking for such information for all 20 behaviors included in the present study could have substantially increased participant fatigue. Based on the preliminary findings we presented, we encourage researchers to further consider how the specific intersections of contexts might affect the antecedents and consequences of people’s willingness (or lack thereof) to engage in sexual activity and their communication of that willingness (or refusal).

## Conclusion

We provided further evidence that people’s willingness, and active communication of that willingness, varies by behavior and by gender. We also added to a growing body of empirical work on sexual consent by broadening the array of sexual behaviors and contexts considered. People are sexually diverse, and their sexual consent is nuanced as a result. Future work on sexual consent should continue to acknowledge and evaluate the variety of sexual behaviors that people engage in.

## Appendix

*List of Diverse Sexual Behaviors*

I have given somebody oral sex (e.g., mouth on genitals)
Somebody has given me oral sex (e.g., mouth on genitals)
I have had vaginal-penile sex
I have stimulated or penetrated somebody’s anus
Somebody has stimulated or penetrated my anus
I have had sex with somebody of a different sex or gender
I have had sex with somebody of the same sex or gender
I have had sex with 2 or more people at the same time (e.g., threesome)
I have had sex with somebody I met online or on a dating app
I have had drunk sex (i.e., under the influence of alcohol)
I have had high sex (i.e., under the influence of other drugs)
I have had sex in public (e.g., car, park, beach)
I have used a sex toy with a partner
I have engaged in sexual role play with somebody
I have engaged in BDSM with somebody (e.g., bondage/discipline, sadism/masochism)
I have sent somebody a sexually explicit picture or video of myself
Somebody has sent me a sexually explicit picture or video of themself
I have had phone or video sex with somebody (e.g., facetime)
I have watched pornography with a sexual partner
I have made a sex tape with somebody

## Note

1. Compared with other platforms (Amazon’s MTurk or CrowdFlower), there is evidence that participants on Prolific Academic are less dishonest, produce higher data quality, are more naïve, fail fewer attention-check questions, and are more diverse (Peer et al., 2017).

## Author Biographies

**Malachi Willis,** PhD, is a research associate in the MRC/CSO Social and Public Health Sciences Unit at the University of Glasgow. He primarily researches the nuances of sexual consent.

**Rebecca Smith,** PhD, is a senior lecturer in psychology for the School of Human Sciences at the University of Greenwich and is affiliated with the Centre for Inequalities within the Institute for Lifecourse Development. Rebecca’s research interests are in all areas of social psychology. Her current research focuses on rape myth acceptance, adult attachment style, and the effects of ostracism.

## References

[bibr1-08862605211044101] Abbey A., Zawacki T., Buck P. O., Clinton A. M., & McAuslan P. (2001). Alcohol and sexual assault. *Alcohol Research & Health*, 25, 43-51. 11496965 PMC4484576

[bibr2-08862605211044101] Barnett M. D., Fleck L. K., Marsden A. D., & Martin K. J. (2017). Sexual semantics: The meanings of sex, virginity, and abstinence for university students. *Personality and Individual Differences*, 106, 203-208. https://doi.org/10.1016/j.paid.2016.11.008

[bibr3-08862605211044101] Beres M. A. (2014). Rethinking the concept of consent for anti-sexual violence activism and education. *Feminism & Psychology*, 24, 373-389. https://doi.org/10.1177/0959353514539652

[bibr4-08862605211044101] Breiding M. J. (2014). Prevalence and characteristics of sexual violence, stalking, and intimate partner violence victimization—National Intimate Partner and Sexual Violence Survey, United States, 2011. *Morbidity and Mortality Weekly Report. Surveillance Summaries*, 63, 1-18. PMC4692457 25188037

[bibr5-08862605211044101] Bryant H. C. (1911). *Ornithology reprints* (Vol. 4). The Ohio State University.

[bibr6-08862605211044101] Campbell R., Dworkin E., & Cabral G. (2009). An ecological model of the impact of sexual assault on women’s mental health. *Trauma, Violence, & Abuse*, 10, 225-246. https://doi.org/10.1177/1524838009334456 10.1177/1524838009334456 19433406

[bibr7-08862605211044101] Chivers M. L. (2005). A brief review and discussion of sex differences in the specificity of sexual arousal. *Sexual and Relationship Therapy*, 20, 377-390. https://doi.org/10.1080/14681990500238802

[bibr8-08862605211044101] Cohen J. (1988). *Statistical power analysis for the behavioral sciences* (2nd ed.). Lawrence Erlbaum Associates.

[bibr9-08862605211044101] Curtis J. N., & Burnett S. (2017). Affirmative consent: What do college student leaders think about “yes means yes” as the standard for sexual behavior? *American Journal of Sexuality Education*, 12, 201-214. https://doi.org/10.1080/15546128.2017.1328322

[bibr10-08862605211044101] Fahs B., & Gonzalez J. (2014). The front lines of the “back door”: Navigating (dis)engagement, coercion, and pleasure in women’s anal sex experiences. *Feminism & Psychology*, 24, 500-520. https://doi.org/10.1177/0959353514539648

[bibr11-08862605211044101] Fanghanel A. (2020). Asking for it: BDSM sexual practice and the trouble of consent. *Sexualities*, 23, 269-286. https://doi.org/10.1177/1363460719828933

[bibr12-08862605211044101] Gidycz C. A., Orchowski L. M., King C. R., & Rich C. L. (2008). Sexual victimization and health-risk behaviors: A prospective analysis of college women. *Journal of Interpersonal Violence*, 23, 744-763. https://doi.org/10.1177/0886260507313944 18272723 10.1177/0886260507313944

[bibr13-08862605211044101] Gidycz C. A., Warkentin J. B., Orchowski L. M., & Edwards K. M. (2011). College men’s perceived likelihood to perpetrate sexual aggression. *Journal of Aggression*, *Maltreatment & Trauma*, 20(03), 260-279. https://doi.org/10.1080/10926771.2011.562480

[bibr14-08862605211044101] Hall D. S. (1998). Consent for sexual behavior in a college student population. *Electronic Journal of Human Sexuality*, 1. http://www.ejhs.org/volume1/consent1.htm

[bibr15-08862605211044101] Hatfield E., Sprecher S., Pillemer J. T., Greenberger D., & Wexler P. (1989). Gender differences in what is desired in the sexual relationship. *Journal of Psychology & Human Sexuality*, 1(02), 39-52. https://doi.org/10.1300/J056v01n02_04

[bibr16-08862605211044101] Herbenick D., Bowling J., Fu T. C., Dodge B., Guerra-Reyes L., & Sanders S. (2017). Sexual diversity in the United States: Results from a nationally representative probability sample of adult women and men. *PloS One*, 12, 1-23. https://doi.org/10.1371/journal.pone.0181198 10.1371/journal.pone.0181198 PMC5519052 28727762

[bibr17-08862605211044101] Herbenick D., Fu T. C., Dodge B., & Fortenberry J. D. (2019). The alcohol contexts of consent, wanted sex, sexual pleasure, and sexual assault: Results from a probability survey of undergraduate students. *Journal of American College Health*, 67(02), 144-152. 29652650 10.1080/07448481.2018.1462827

[bibr18-08862605211044101] Hickman S. E., & Muehlenhard C. L. (1999). “By the semi‐mystical appearance of a condom”: How young women and men communicate sexual consent in heterosexual situations. *Journal of Sex Research*, 36, 258-272. https://doi.org/10.1080/00224499909551996

[bibr19-08862605211044101] Humphreys T. (2007). Perceptions of sexual consent: The impact of relationship history and gender. *Journal of Sex Research*, 44, 307-315. https://doi.org/10.1080/00224490701586706 18321010 10.1080/00224490701586706

[bibr20-08862605211044101] Jozkowski K. N., & Peterson Z. D. (2013). College students and sexual consent: Unique insights. *Journal of Sex Research*, 50(06), 517-523. https://doi.org/10.1080/00224499.2012.700739 23039912 10.1080/00224499.2012.700739

[bibr21-08862605211044101] Jozkowski K. N., Sanders S., Peterson Z. D., Dennis B., & Reece M. (2014). Consenting to sexual activity: The development and psychometric assessment of dual measures of consent. *Archives of Sexual Behavior*, 43, 437-450. https://doi.org/10.1007/s10508-013-0225-7 24452630 10.1007/s10508-013-0225-7

[bibr22-08862605211044101] Jozkowski K. N., & Wiersma J. D. (2015). Does drinking alcohol prior to sexual activity influence college students’ consent? *International Journal of Sexual Health*, 27, 156-174. https://doi.org/10.1080/19317611.2014.951505

[bibr23-08862605211044101] King B. M., Fallon M. R., Reynolds E. P., Williamson K. L., Barber A., & Giovinazzo A. R. (2021). College students’ perceptions of concurrent/successive nonverbal behaviors as sexual consent. *Journal of Interpersonal Violence*, 36(23–24), NP13121-NP13135. https://doi.org/10.1177/0886260520905544 10.1177/0886260520905544 32052687

[bibr24-08862605211044101] Koss M. P., Abbey A., Campbell R., Cook S., Norris J., Testa M., & White J. (2007). Revising the SES: A collaborative process to improve assessment of sexual aggression and victimization. *Psychology of Women Quarterly*, 31, 357-370. https://doi.org/10.1111/j.1471-6402.2007.00385.x

[bibr25-08862605211044101] Marcantonio T., Jozkowski K. N., & Wiersma-Mosley J. (2018). The influence of partner status and sexual behavior on college women’s consent communication and feelings. *Journal of Sex & Marital Therapy*, 44, 776-786. https://doi.org/10.1080/0092623X.2018.1474410 29741449 10.1080/0092623X.2018.1474410

[bibr26-08862605211044101] Mark K. P., & Vowels L. M. (2020). Sexual consent and sexual agency of women in healthy relationships following a history of sexual trauma. *Psychology & Sexuality*, 4, 315-328. https://doi.org/10.1080/19419899.2020.1769157

[bibr27-08862605211044101] Maynard E., Carballo-Diéguez A., Ventuneac A., Exner T., & Mayer K. (2009). Women’s experiences with anal sex: motivations and implications for STD prevention. *Perspectives on Sexual and Reproductive Health*, 41, 142-149. https://doi.org/10.1363/4114209 19740231 10.1363/4114209 PMC5588879

[bibr28-08862605211044101] Michel J. B., Shen Y. K., Aiden A. P., Veres A., Gray M. K., Brockman W., & Aiden E. L. (2011). Quantitative analysis of culture using millions of digitized books, *Science*, 331, 176-182. https://doi.org/10.1126/science.1199644 21163965 10.1126/science.1199644 PMC3279742

[bibr29-08862605211044101] Milhausen R. R., Graham C. A., Sanders S. A., Yarber W. L., & Maitland S. B. (2010). Validation of the sexual excitation/sexual inhibition inventory for women and men. *Archives of Sexual Behavior*, 39(05), 1091-1104. https://doi.org/10.1007/s10508-009-9554-y 19859799 10.1007/s10508-009-9554-y

[bibr30-08862605211044101] Mori C., Temple J. R., Browne D., & Madigan S. (2019). Association of sexting with sexual behaviors and mental health among adolescents: A systematic review and meta-analysis. *JAMA Pediatrics*, 173, 770-779. https://doi.org/10.1001/jamapediatrics.2019.1658 31206151 10.1001/jamapediatrics.2019.1658 PMC6580450

[bibr31-08862605211044101] Muehlenhard C. L., Humphreys T. P., Jozkowski K. N., & Peterson Z. D. (2016). The complexities of sexual consent among college students: A conceptual and empirical review. *Journal of Sex Research*, 53, 457-487. https://doi.org/10.1080/00224499.2016.1146651 27044475 10.1080/00224499.2016.1146651

[bibr32-08862605211044101] Opperman E., Braun V., Clarke V., & Rogers C. (2014). “It feels so good it almost hurts”: Young adults’ experiences of orgasm and sexual pleasure. *Journal of Sex Research*, 51, 503-515. https://doi.org/10.1080/00224499.2012.753982 23631739 10.1080/00224499.2012.753982

[bibr33-08862605211044101] Peer E., Brandimarte L., Samat S., & Acquisti A. (2017). Beyond the Turk: Alternative platforms for crowdsourcing behavioral research. *Journal of Experimental Social Psychology*, 70, 153-163. https://doi.org/10.1016/j.jesp.2017.01.006

[bibr34-08862605211044101] Peterson Z. D., & Muehlenhard C. L. (2007). Conceptualizing the “wantedness” of women’s consensual and nonconsensual sexual experiences: Implications for how women label their experiences with rape. *Journal of Sex Research*, 44, 72-88. https://doi.org/10.1080/00224490709336794 17599266 10.1080/00224490709336794

[bibr35-08862605211044101] Pinsky H. T., Shepard M. E., Bird E. R., Gilmore A. K., Norris J., Davis K. C., & George W. H. (2017). Differences in mental health and sexual outcomes based on type of nonconsensual sexual penetration. *Violence Against Women*, 23, 1039-1054. https://doi.org/10.1177/1077801216655624 27486127 10.1177/1077801216655624 PMC5225245

[bibr36-08862605211044101] Righi M. K., Bogen K. W., Kuo C., & Orchowski L. M. (2019). A qualitative analysis of beliefs about sexual consent among high school students. *Journal of Interpersonal Violence*, 36, 1-27. https://doi.org/10.1177/0886260519842855 10.1177/0886260519842855 30973037

[bibr37-08862605211044101] Rueff W. T., & Gross A. M. (2017). Assessing sexual coercion: Survey wording differences and the victimization-perpetration discrepancy. *Journal of Family Violence*, 32, 325-331. https://doi.org/10.1007/s10896-016-9859-2

[bibr38-08862605211044101] Sabina C., & Straus M. A. (2008). Polyvictimization by dating partners and mental health among US college students. *Violence and Victims*, 23, 667-682. https://doi.org/10.1891/0886-6708.23.6.667 19069560 10.1891/0886-6708.23.6.667

[bibr39-08862605211044101] Sanders S. A., Hill B. J., Yarber W. L., Graham C. A., Crosby R. A., & Milhausen R. R. (2010). Misclassification bias: Diversity in conceptualisations about having “had sex”. *Sexual Health*, 7, 31-34. https://doi.org/10.1071/SH09068 20152093 10.1071/SH09068

[bibr40-08862605211044101] Strang E., Peterson Z. D., Hill Y. N., & Heiman J. R. (2013). Discrepant responding across self-report measures of men’s coercive and aggressive sexual strategies. *Journal of Sex Research*, 50, 458-469. https://doi.org/10.1080/00224499.2011.646393 22329465 10.1080/00224499.2011.646393

[bibr41-08862605211044101] Walsh K., Honickman S., Valdespino-Hayden Z., & Lowe S. R. (2019). Dual measures of sexual consent: A confirmatory factor analysis of the Internal Consent Scale and External Consent Scale. *Journal of Sex Research*, 56, 802-810. https://doi.org/10.1080/00224499.2019.1581882 30882249 10.1080/00224499.2019.1581882

[bibr42-08862605211044101] Willis M., & Jozkowski K. N. (2018). Barriers to the success of affirmative consent initiatives: An application of the Social Ecological Model. *American Journal of Sexuality Education*. 12, 324-336. https://doi.org/10.1080/15546128.2018.1443300

[bibr43-08862605211044101] Willis M., & Jozkowski K. N. (2019). Sexual precedent’s effect on sexual consent communication. *Archives of Sexual Behavior*, 48, 1723-1734. https://doi.org/10.1007/s10508-018-1348-7 31016491 10.1007/s10508-018-1348-7

[bibr44-08862605211044101] Willis M., Blunt-Vinti H. D., & Jozkowski K. N. (2019). Assessing and addressing the need for more diverse samples regarding age and race/ethnicity in sexual consent research. *Personality and Individual Differences*, 149, 37-45. https://doi.org/10.1016/j.paid.2019.05.029

[bibr45-08862605211044101] Willis M., Hunt M., Wodika A., Rhodes D. L., Goodman J., & Jozkowski K. N. (2019). Explicit verbal sexual consent communication: Effects of gender, relationship status, and type of sexual behavior. *International Journal of Sexual Health*, 31, 60-70. https://doi.org/10.1080/19317611.2019.1565793

[bibr46-08862605211044101] Willis M., Jozkowski K. N., Bridges A. J., Veilleux J. C., & Davis R. E. (2021a). Assessing the within-person variability of internal and external sexual consent. *Journal of Sex Research*, 58(9), 1173-1183. https://doi.org/10.1080/00224499.2021.1913567 33929282 10.1080/00224499.2021.1913567 PMC9239691

[bibr47-08862605211044101] Willis M., Marcantonio T. L., & Jozkowski K. N. (2021b). Internal and external sexual consent during events that involved alcohol, cannabis, or both. *Sexual Health*, 18, 260-268. https://doi.org/10.1071/SH21015 34134817 10.1071/SH21015

[bibr48-08862605211044101] Willis M., Jozkowski K. N., Bridges A. J., Davis R. E., & Veilleux J. C. (2021c). Developing valid and feasible measures of sexual consent for experience sampling methodology. *Journal of Sex Research*, 58, 996-1007. https://doi.org/10.1080/00224499.2021.1907526 33891521 10.1080/00224499.2021.1907526 PMC9239692

